# Development and evaluation of a pre-clerkship spiral curriculum: data from three medical school classes

**DOI:** 10.1080/10872981.2023.2167258

**Published:** 2023-01-15

**Authors:** Anthony J. Maltagliati, Joshua H. Paree, Kadian L. McIntosh, Kevin F. Moynahan, Todd W. Vanderah

**Affiliations:** The University of Arizona College of Medicine, 1501 N Campbell Ave, Tucson, AZ, USA

**Keywords:** Medical education, pre-clerkship, spiral curriculum, time-spaced repetition, medical curriculum

## Abstract

Pre-clerkship curricula of most Liaison Committee on Medical Education (LCME)-accredited medical schools are divided into blocks by organ system, leaving a significant amount of information susceptible to loss due to prolonged nonuse. We describe the implementation of a formal Spiral Curriculum that periodically revisits material from previous blocks. Learners were surveyed on receptivity to the curriculum across three graduating classes at a single medical school. Medical school graduate classes of 2020, 2021, and 2022 were surveyed at the end of their pre-clerkship years (2018–2020). The class of 2022 actually received the Spiraled Curriculum intervention, for which the authors created 500 board exam style multiple-choice questions, periodically administered via mandatory in-class sessions ranging from 10 to 20 questions reviewing content from previous blocks with designated expert faculty. Response rates were 36% (*n* = 46), 45% (*n* = 52), and 32% (*n* = 40) for classes of 2020, 2021, and 2022, respectively. On a Likert scale (1 = strongly disagree, 5 = neutral, 10 = strongly agree), the classes of 2020, 2021, and 2022 provided statistically significant differences in their belief that a Spiraled Curriculum would/did help them retain information as 8.2 (SD 1.7), 8.2 (SD 2.2), and 5.0 (SD 3.0) (*n* < 0.05). All classes endorsed neutral confidence in the existing pre-clerkship curriculum in themselves to prepare for United Stated Medical Licensing Examination (USMLE) Step 1, and in their retention of previous block material with no statistically significant differences between classes. USMLE Step 1 scores did not differ significantly between classes (*n* = 0.21). Those who did not receive the Spiral Curriculum were highly receptive to it in theory, while those who actually received the intervention gave a neutral rating. Per survey comments, implementation of a Spiraling Curriculum would ideally be administered as either team-based or self-directed activities, and a Spiraling Curriculum may be difficult to implement in accelerated (18 month) pre-clerkship formats.

**Practice points**

- Question: What is the receptivity of medical students to a formal Spiral curriculum that uses time-spaced repetition sessions of board exam style questions to revisit previous block content of their pre-clerkship years?
- Findings: In this single-center, quasi-experimental study, the two control group medical school classes had very positive theoretical reception to a Spiral curriculum proposal (rated 8 out of 10) while the class who actually received the Spiral curriculum provided a statistically significant lower neutral rating (rated 5 out of 10), citing preference for a team-based or self-directed format.
- Meaning: Medical students are strongly in favor of structured time-spaced repetition with board exam style questions to revisit previous material but prefer a format that does not interfere with time to personalize their medical school experience.

## Introduction

The volume of information conveyed throughout medical school is immense, with the pre-clerkship curricula of 88% of Liaison Committee on Medical Education-accredited (LCME) medical schools opting to organize content and assessments into blocks by organ system [1]. When content is divided into blocks, information loss due to prolonged nonuse has been estimated as ‘around 70% retention after one year, 40–50% after two years, and 30% after four years or more’ [2]. Time spaced repetition is posited as a way to improve learning efficiency and information retention, with evidence spanning from cellular signaling and neuroplasticity to the behavioral level [2,3]. This would result in a curriculum that is not linear over time but circles back on itself as the knowledge base broadens, like a spiral [4,5]. Examples of spiral curricula pertaining to single areas of focus in medical or pharmacy school such as concussions, oral health, dyspepsia, and leadership have been published, but the authors are unaware of any curricular innovation implementation on the scope and scale described herein [6–9]. Furthermore, the discrepancy of formal medical school education and a self-directed medical student curriculum is well described, with a need for medical schools to reconcile presenting their curricula in a practical and relevant format to increase both the perceived value to and engagement of medical students [10].

Whether medical schools intentionally implement periodic review of previous content, as well as what methodology is used for such reviews, is not currently categorized or differentiated by the Association of American Medical Colleges reports of medical school curricula [1]. A 2017 study found that medical students at one public institution engaged in a self-directed ‘parallel’ Step 1 curriculum using third-party study resources, and several study behaviors were associated with improved USMLE Step 1 performance (even when controlling for Medical College Admission Test (MCAT) scores, preclinical exam performance, and self-identified score goal), including initiating Step 1 study prior to the designated study period, increased review book usage, and attempting more practice questions [11]. Given the benefit of an unofficial parallel curriculum, we aimed to ascertain whether a formal medical school curriculum that revisits high-yield material from previous blocks will have a similar benefit and be well received by students. To investigate the practicality and receptivity to such a strategy, we developed a ‘Pre-Clerkship Spiral Curriculum’ that periodically revisits material from previous blocks using board exam style multiple-choice questions.

## Methods

Three medical school classes were surveyed anonymously and voluntarily prior to their dedicated Step 1 study period using a Likert scale (1 = strongly disagree, 5 = neutral, 10 = strongly agree) to assess perceptions of the pre-clerkship phase of medical school and receptivity to a Spiral Curriculum, with the classes of 2020 and 2021 serving as control groups and the class of 2022 actually receiving the intervention. Statistical analysis was performed with ANOVA on Microsoft Excel. We created 500 multiple-choice board exam style questions that were vetted by faculty with expertise in their respective fields. On weeks with no examinations during their 18-month pre-clerkship phase of medical school, students in the class of 2022 were required to attend 1-hour in-class assessment sessions ranging from 10 to 20 questions taken individually on electronic devices, with total time allowed equated to 90 seconds per question. Questions covered material from previous blocks (horizontal learning), became gradually more difficult and comprehensive (vertical learning), and were used purely for self-assessment rather than part of a grade. Immediately following assessments, students received their scores and reviewed questions with expert faculty via slides and written explanations. There were 32 sessions total (Table 1) with sessions (not in order) including musculoskeletal (4), neurology (4), pharmacology (3), histology/pathology (3), cell biology (2), biochemistry (2), microbiology (2), cardiology/pulmonary/renal (C/P/R) (2), digestion/metabolism/hormones (D/M/H) (2), biochemistry (2), biostatistics (2), psychiatry (2), genetics (1), and dermatology (1).

**Table 1.** Spiral Curriculum and block schedule for class of 2022.

Spiral Curriculum Schedule				
Dates	Block	Date	Session Number	Session Content
07/09/2018–07/20/2018	Bridge to Medical School (optional)	–	–	–
07/23/2018–07/27/21018	Orientation Week	–	–	–
07/30/2018–09/07/2018	Foundations	07/30/2018	1	Cell Biology
		08/06/2018	2	Biochemistry
		08/13/2018	3	Biostatistics
		08/20/2018	4	Histology
		08/27/2018	5	Pharmacology
09/10/2018–10/19/2018	Musculoskeletal	09/17/2018	6	Histology
		09/24/2018	7	Pathology
		10/08/2018	8	Immunology
		10/15/2018	9	Pharmacology
10/22/2018–12/21/2018	Nervous	10/22/2018	10	Biochemistry
		10/29/2018	11	Microbiology
		11/05/2018	12	Musculoskeletal
		11/26/2018	13	Musculoskeletal
		12/10/2018	14	Dermatology
12/24/2018–1/04/2019	Winter Recess	–	–	–
01/07/2019–03/22/2019	Cardiovascular, Pulmonary, Renal (C/P/R)	01/07/2019	15	Cell Biology
		01/14/2019	16	Neurology
		01/28/2019	17	Microbiology
		02/18/2019	18	Neurology
		03/04/2019	19	Musculoskeletal
		03/11/2019	20	Musculoskeletal
03/25/2019–03/29/2019	Spring Recess	–	–	–
04/01/2019–05/31/2019	Digestion, Metabolism, Hormones (D/M/H)	04/08/2019	21	Biostatistics
		04/15/2019	22	Psychiatry
		05/06/2019	23	Genetics
		05/13/2019	24	C/P/R
		05/20/2019	25	Immunology
06/03/2019–08/09/2019	Summer Recess	–	–	–
08/12/2019–09/27/2019	Life Cycle	08/12/2019	26	Psychiatry
		08/19/2019	27	Neurology
		08/26/2019	28	D/M/H
		09/16/2019	29	Neurology
09/30/2019–11/22/2019	Immunology & Infection	10/07/2019	30	C/P/R
		10/21/2019	31	D/M/H
		11/18/2019	32	Pharmacology
11/25/2019–12/20/2019	Advanced Topics	–	–	–
12/23/2019–01/03/2020	Winter Recess	–	–	–
01/06/2020–02/21/2020	Dedicated USMLE Step 1 Study	–	–	–

## Results

Baseline characteristics of the classes of 2020, 2021, and 2022 (Table 2A) were obtained from public admissions records as averages, which precluded further analysis for statistical significance. Observed trends include a gradual increase in the average age, overall grade point average (GPA), science GPA, and MCAT percentile over the three-year span. Of note the class of 2020 was administered a 24-month pre-clerkship curriculum while the classes of 2021 and 2022 were 18 months.

**Table 2.** Baseline characteristics of UACOM-T medical school classes (2A). Survey responses and USMLE Step 1 class averages from the classes of 2020, 2021, and 2022 (2B).

A. Baseline Characteristics of UACOM-T Medical School Classes				
Characteristic	Class of 2020 n = 132	Class of 2021 n = 120	Class of 2022 n = 125	
Age, average (min-max)	22 (20–39)	25 (21–48)	25 (21–35)	
Males, n (%)	58 (44)	65 (54)	60 (48)	
URM, n (%)	46 (35)	37 (31)	39 (31)	
In-state, n (%)	88 (67)	80 (67)	93 (74)	
GPA overall, average	3.58	3.65	3.76	
GPA science, average	3.48	3.54	3.56	
MCAT, average (percentile)	Old: 28.4 (70) New: 504.8 (70)	Old: 27.7 (64) New: 507 (79)	Old: N/A New: 507 (79)	
Pre-clerkship curriculum length	24 months	18 months	18 months	
B. Survey Responses & USMLE Step 1 Averages				
Survey Prompt	Class of 2020 n = 46 (36%)	Class of 2021 n = 52 (45%)	Class of 2022 n = 40 (32%)	*p*-value
Confident curriculum prepared me for Step 1	5.2, SD 2.2	5.6, SD 2.0	5.6, SD 2.0	0.55
Curriculum decreased stress level regarding Step 1	4.2, SD 2.5	4.1, SD 2.0	4.4, SD 2.1	0.73
Feel I have retained majority of material from previous blocks	5.0, SD 2.4	4.6, SD 2.0	4.2, SD 1.9	0.15
Confident to obtain my goal score for Step 1	6.2, SD 2.2	6.1, SD 2.3	5.6, SD 2.0	0.33
Hours per week studying for Step 1 prior to dedicated study period	4–6, SD 1.6	4–6, SD 1.5	4–6, SD 1.5	0.83
I have had adequate time to tend to wellness	7.2, SD 2.4	5.3, SD 2.3	4.8, SD 2.5	<0.005
I believe a Spiral Curriculum would/did help me retain material as I progressed through blocks	8.2, SD 1.6	8.2, SD 2.2	5.0, SD 2.9	<0.000005
USMLE Step 1 Score, class average (SD)	224 (18)	229 (21)	227 (20)	0.21

**Figure 1.**
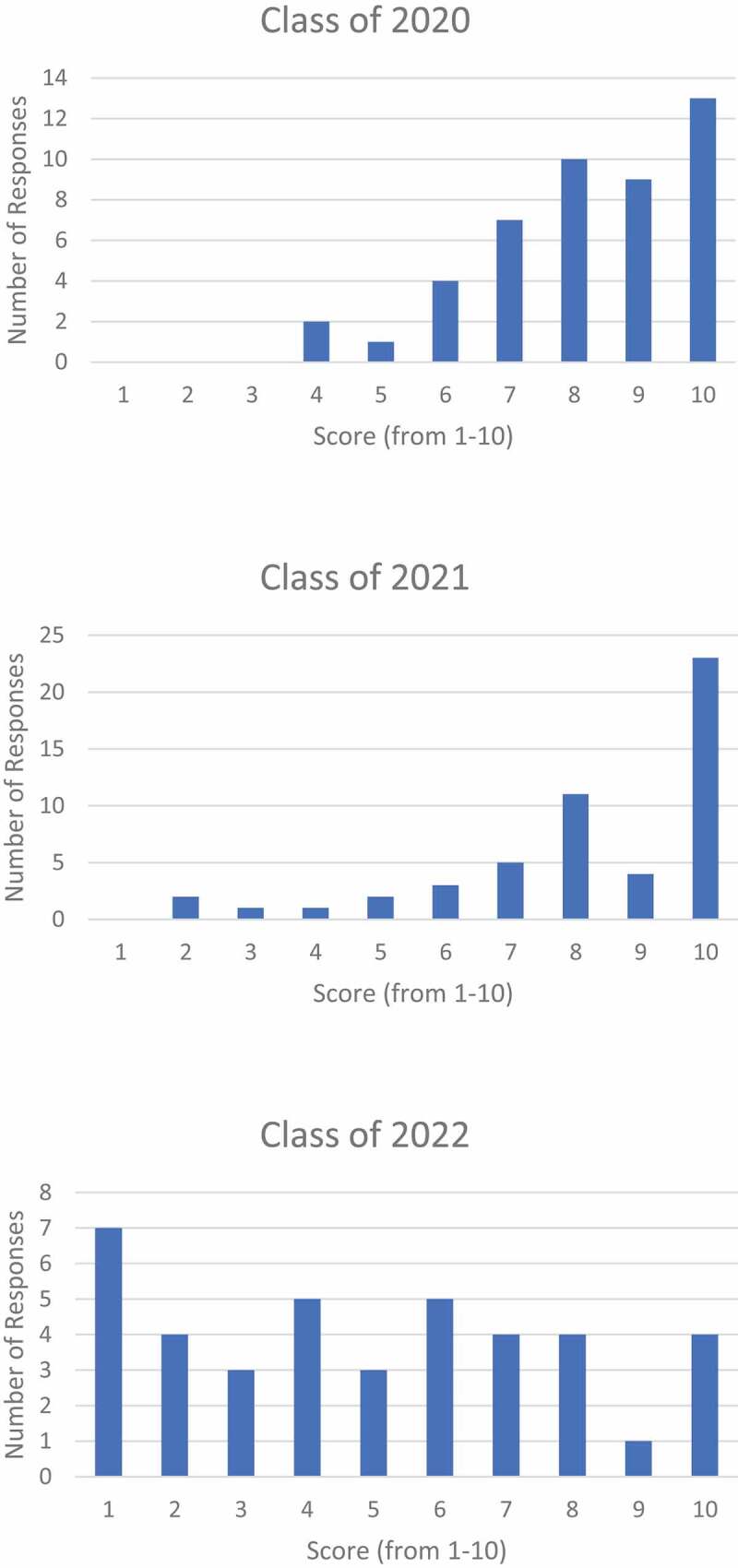
Response distribution to prompt ‘I believe a Spiral Curriculum would/did help me retain material as I progressed through blocks’ with Classes of 2020 and 2021 rating hypothetically and Class of 2022 rating after receiving the Spiral Curriculum.

Survey response rates for graduate classes of 2020, 2021, and 2022 were 36% (*n* = 46), 45% (*n* = 52), and 32% (*n* = 40), respectively. The classes of 2020, 2021, and 2022 provided statistically significant differences (*n* < 0.05) in their responses to perceived time for wellness (2020: 7.2, SD 2.4; 2021: 5.3, SD 2.3; 2022: 4.8, SD 2.5), with lower perceived time for wellness in the classes administered a shorter, ‘accelerated’ (18 month) pre-clerkship curriculum. Statistically significant differences (*n* < 0.05) also resulted regarding receptivity to the Spiraled Curriculum (2020: 8.2, SD 1.6; 2021: 8.2, SD 2.2; 2022: 5.0, SD 2.9), with the classes who did not receive the intervention viewing it favorably as a hypothetical proposal and the class who actually received the spiral curriculum providing a neutral rating (Figure 1). Average USMLE Step 1 scores did not differ with statistical significance (*n* = 0.21; Table 2B).

Each class exhibited different trends in the survey optional comment section (class of 2020 *n* = 19; class of 2021 *n* = 13; class of 2022 *n* = 11) summarized below:

- Class of 2020: studying for block exams and Step 1 felt like two different tasks; wished to have more incorporated board studying.
- Class of 2021: supported longitudinal time-spaced review but concern the accelerated 18-month curriculum (first implemented with this class) may be too fast-paced to review previous block material.
- Class of 2022: prefer Spiral Curriculum as self-directed learning; mandatory attendance was viewed as inconvenient and precluded other academic pursuits and/or wellness.

## Discussion

Our study shows the surveyed medical students perceive a pre-clerkship Spiral Curriculum favorably in theory but had a neutral response upon actual implementation. Survey comments indicate this was most attributable to the Spiral Curriculum being administered via mandatory in-class sessions, with a preference for a self-directed format instead. Specifically, respondents expressed they wish to maximize time for wellness and/or extracurricular pursuits. The authors posit the sentiment of medical students will be translatable across institutions given self-directed and other learning formats outside of the traditional lecture format are highly utilized to supplement if not almost entirely replace formal curricula [10]. As nearly 90% of LCME-accredited medical schools use a block format for their pre-clerkship curricula, the design of this study has the benefit of being generalizable and relevant to most medical schools.

A Spiral Curriculum design based on adult learning principles could be the solution for both an unmet need of medical schools to increase student interest in their curricula as well as formally require medical students to routinely review and integrate content over the course of their pre-clerkship studies to integrate organ systems and minimize attrition of knowledge. Key elements of adult learning in cognitive science that were sought in developing this Spiral Curriculum include spaced repetition – or periodic revisiting of material – as well as ‘active recall’ and the ‘testing effect’ that demonstrate answering test questions with feedback (i.e., scores and explanations) is more engaging than simply re-reading or receiving a lecture review session on a topic [12,13]. By featuring and supplying an additional 500 multiple choice board exam style questions to students as part of their education, both the faculty and students can conceivably identify gaps in knowledge and the extent of these gaps in a more uniform manner across a medical school class. Ideally this could lead to both personalized study plans for students and reveal areas for improvement for educators in an unambiguous manner. Furthermore, it could decrease the financial burden faced by medical students who might otherwise feel compelled to invest in more third-party resources (i.e., question banks) than are necessary. The optimal interval for spaced repetition has recently been shown to be individualized but amenable to modeling [14], and a self-directed Spiral Curriculum may be the first of many steps in creating a formal medical school education paradigm that is increasingly tailored to each learner. It is worth noting that accelerated 18-month pre-clerkship curricular formats were associated with a statistically significantly lower perceived time for wellness among survey respondents. The authors posit this association further makes a case for self-directed formats of medical education, particularly for self-assessment sessions in accelerated curricula such as those administered in the Spiral Curriculum described here.

The closest comparison to this work in terms of scope and principle comes from a 2022 paper out of the Netherlands [15], wherein previously tested content was incorporated into each major block exam as students progressed. Overall, the comprehensive aforementioned study with a dataset spanning five years showed time-spaced repetition mitigates loss of medical knowledge, but too much emphasis on reviewing past material may slightly lower overall scores when tested on both new and old material simultaneously. There are several fundamental differences between that study and our Spiral Curriculum: The Spiral Curriculum questions did not contribute to a grade but rather served solely as a self-assessment; Spiral Curriculum sessions were standalone sessions that were purposely not assigned on examination weeks to allow learners to focus on upcoming examinations, and Spiral Curriculum sessions were immediately followed by a comprehensive review of each question hosted by various expert faculty. While there are parallels of employing time-spaced repetition, active recall, and the ‘testing effect’ to improve knowledge retention and integration over time, the critical differences in design and implementation make the research questions and conclusions of each work related but distinct.

Limitations of this study include a single-center study with a modest survey response rate. Multiple possible confounding variables, including different baseline characteristics of each class and different pre-clerkship curriculum lengths, preclude a conclusion as to what extent a pre-clerkship Spiral Curriculum impacted Step 1 scores. The USMLE decision to change Step 1 to pass/fail may alleviate some of the stress associated with the exam, but the authors postulate this change does not eliminate the need for knowledge retention over time or the importance of building strong neural networks and a fund of knowledge in the formative pre-clerkship years.

Future directions for this work include statistical analysis using Pearson correlation to gauge the relationship of each learner’s scores on Spiral Curriculum questions with graded examinations and USMLE Board examinations to determine if the Spiral Curriculum may be a useful predictor for performance on high-stakes block and board examinations. Additionally, analysis to determine the mean and standard deviation of each session as well item analysis for each multiple-choice question’s difficulty (percentage correct) and discrimination (correlation of responses to individual items with overall test score) will highlight opportunities to revise and improve the multiple-choice questions themselves and/or identify gaps in the way the material is taught by faculty. Repeating the survey for the Class of 2023 and beyond, who will have had the Spiral Curriculum administered virtually due to the COVID-19 pandemic, will provide additional data to adequately power the analyses as well as provide valuable insight and context for how the Spiral Curriculum was received in a remote learning setting. Finally, our group intends to weigh the feasibility of a Spiral Curriculum for the clerkship year(s) of medical school with the intent of revisiting and integrating high-yield material across core clerkships and preparing medical students for USMLE Step 2.

In summary, those who did not receive the Spiral Curriculum were highly receptive to it in theory, while those who received the intervention gave a neutral rating. Per survey comments, implementation of a Spiraling Curriculum would ideally be administered as either team-based or self-directed activities, and a Spiraling Curriculum may be difficult to implement in accelerated (18 month) pre-clerkship formats. The feedback and lessons learned while designing and implementing this innovation are relevant and translatable to other institutions when considering any similar changes to their curriculum. The authors hope this may serve as a valuable resource for institutions who pursue their own renditions of a Spiral Curriculum or other time-spaced repetition learning strategies.

## Ethical Approval

This work was approved as a sub-study under ‘A Longitudinal Study of Medical Education Processes and Outcomes’ at the University of Arizona College of Medicine – Tucson, for which Dr. Kevin Moynahan serves as the Primary Investigator.

Access to data and data analysis: Maltagliati AJ and Paree JH conducted the analysis on anonymous or completely deidentified data. Vanderah TW had full access to all the data in the study and takes responsibility for the integrity of the data and the accuracy of the data analysis.

Meeting presentation: a brief summary of the study rationale, design, and preliminary survey data was presented as a top five submission for ‘Innovations in Undergraduate Medical Education’ at the Innovations in Medical Education Conference hosted by the University of Southern California Keck School of Medicine on 14 February 2020 in Los Angeles, CA.

## Originality of content

All information and materials in the manuscript are original.

## Study type

Non-randomized, controlled trial/survey study.
